# Non-infectious skeletal complications in the lower extremity after treatment with a transfemoral bone-anchored prosthesis: a retrospective observational study

**DOI:** 10.1136/bmjopen-2025-105415

**Published:** 2026-02-24

**Authors:** Karin Svensson Malchau, Henrik Malchau, Peter Thomsen, Kerstin Hagberg

**Affiliations:** 1 Department of Orthopaedics, Institute of Clinical Sciences, University of Gothenburg, Sahlgrenska Academy, Gothenburg, Sweden; 2 Department of Orthopaedics, Sahlgrenska University Hospital, Gothenburg, Sweden; 3 Department of Biomaterials, University of Gothenburg Institute of Clinical Sciences, Gothenburg, Sweden; 4 Department of Biomedical Dental Sciences, Imam Abdulrahman Bin Faisal University, Dammam, Saudi Arabia

**Keywords:** Lower Extremity, Limb reconstruction, Orthopedics, Amputation, Surgical, Fractures, Bone

## Abstract

**Abstract:**

**Background:**

The use of bone-anchored prostheses (BAPs) has greatly increased quality of life for lower limb amputees. However, the long-term frequency of skeletal fractures and the need for arthroplasty surgery in the lower extremities following BAP use is scarce.

**Objectives:**

The current study aimed to investigate the frequency of fractures and arthroplasties in the lower limb after BAP surgery with the Osseointegrated Prosthesis for the Rehabilitation of Amputees (OPRA) system.

**Design:**

Retrospective cohort study using the OPRA database and medical record review for data collection.

**Setting:**

A single-centre study at a tertiary hospital.

**Participants:**

All patients with a transfemoral BAP (OPRA system) who underwent surgery between 1999 and 2019, and had completed at least 2 years of follow-up were included in the study. Patients with bilateral transfemoral amputations were excluded. A total of 100 patients were included.

**Primary outcome measure:**

The primary outcome measure was to identify patients who had a fracture or had undergone arthroplasty surgery in the lower extremities after BAP surgery.

**Results:**

Of the 100 patients included, 16 patients (16%) had an event in their lower limb. 11 patients (11%) had a fracture, all of the femur, and six patients (6%) underwent arthroplasty surgery due to osteoarthritis. Long-term prosthetic use was not affected by the occurrence of an event.

**Conclusions:**

Patients with BAP may be at a higher risk for femur fractures and arthroplasty surgery than the general population. Although encouraging that prosthetic usage is not affected after a fracture or arthroplasty surgery, prospective studies on larger cohorts and control groups need to be conducted.

**STRENGTHS AND LIMITATIONS OF THIS STUDY**

- The current study includes a cohort of 100 consecutive patients making it a study of one of the largest bone-anchored prostheses (BAP) cohorts.
- This is the first study on BAPs with up to 20 years follow-up.
- All surgeries, rehabilitation and follow-ups were conducted in a standardised manner at the study site.
- A greater number of patients may have been affected by osteoarthritis (OA) and a limitation in the current study is that radiographs were not conducted to evaluate the prevalence of OA, thereby only patients eligible for arthroplasty surgery were identified as a marker of OA.
- Patients resided in different European countries and although events were assessed during follow-ups, access to medical records was limited to patients residing in the same region as the study site.

## Introduction

 Osseointegration was first described in the 1950s and implies the structural connection between the implant and bone.^1^ This discovery paved the way for the use of bone-anchored prostheses (BAP) for clinical use. In orthopaedics, transcutaneous BAPs are used in patients who have undergone amputations, often due to trauma or tumours, who have experienced severe problems functioning with a socket-mounted type artificial limb. The recent advances within this medical field have enabled amputees to regain physical function with an artificial limb without the pain and skin-related problems a socket prosthesis may imply.^2 3^ One such implant system is the Osseointegrated Prosthesis for the Rehabilitation of Amputees system (OPRA Implant System) which has been in use since the 1990s.^4 5^

The OPRA system consists of three main parts: a bone-anchored fixture in which the percutaneous abutment is connected through the abutment screw. Thereafter, an external prosthesis can be attached via a safety device, so-called Axor II, which protects the osseointegrated implant parts if the external prosthesis is made subject to great forces. The OPRA system requires two surgeries: stage 1 (S1) for fixation of the fixture, and stage 2 (S2) 3–6 months later for insertion of the abutment once the fixture has been integrated in bone.^6^ Rehabilitation is commenced after S2 with a gradual and structured increase of weight bearing and further rehabilitation during 6–12 months postoperatively.^7 8^

Percutaneous BAPs, such as the OPRA system, have substantially improved the functional outcome and quality of life for many patients with amputations.^9^ The OPRA system has been described as revolutionary in the life of amputees,^10^ and treated patients have reported increased prosthetic usage, also in the long term.^11^ Other implant systems used in patients with lower limb amputations have been evaluated with similar increases in prosthetic usage.^12 13^

However, there are issues with mechanical and non-infectious complications after percutaneous BAP. As there is great stress at the fixture-abutment site, exchange of the abutment and/or abutment screw occurred in 29% of patients with an OPRA system due to implant fractures 5 years postoperatively.^5 14^ Moreover, prospective studies of the transfemoral OPRA implant system have revealed a 17% revision-free survival rate for mechanical complications after 10 years.^11^ Further, individuals with lower limb amputations are more prone to develop osteoarthritis (OA) in the longer term of the contralateral hip and knee,^15^ and osteoporosis in the ipsilateral extremity.^16^,^18^

The OPRA system is considered a stable system with well-fixated implants.^19^ As patient satisfaction among those treated with BAP devices is high as compared with their previous situation using conventional socket prostheses, the need for this kind of implant system may increase in the future.^4^ Nevertheless, the frequencies of fractures and arthroplasty surgery in the lower extremities following long-term OPRA treatment remain unknown. In the present study, we aimed to investigate the frequency of femoral fractures and arthroplasties, as well as the impact of these complications on the prosthetic use after OPRA surgery.

## Methods and materials

All patients registered with an OPRA Implant System due to a transfemoral amputation (TFA) in the OPRA database at the Sahlgrenska University hospital (SU) were screened. Patients who had their S1 between 31 December 1998 and 31 December 2019, and who had completed their treatment (S1, S2 and rehabilitation) at SU were included in the study. Patients operated on with an OPRA system at SU were invited to standardised follow-ups at 1, 2, 3, 5, 7, 10, 15 and 20 years after treatment.

Medical records were reviewed and assessed until 31 December 2021. Patients who did not have at least 2 years follow-up at that time point were excluded. Further exclusion criteria were missing consent and the presence of bilateral TFAs. Bilateral TFAs were excluded as these patients were considered to have a different risk for events following surgery.

Medical records were reviewed, and the following variables were retrieved: age, sex, body mass index, American Society of Anesthesiologists Physical Status score, laterality, date of amputation, cause of amputation, number of fixture revisions, new injuries in the lower extremity and mortality. Complete medical records were available for patients from the Western Region in Sweden (n=9, 9%) and, in remaining cases (other regions in Sweden (n=52, 52%), other European citizenship (n=39, 39%), complications, if any, were disclosed by the patient during follow-ups organised by the Sahlgrenska University multidisciplinary treatment team. Any experience of a fracture or arthroplasty surgery in the lower extremities was referred to as an event. To evaluate prosthetic usage, the Q-TFA Prosthetic Use Score (PUS, 0–100) was retrieved from the database. The PUS is a patient-reported outcome combining the number of days/week and number of hours per day the prosthesis is normally used. A figure of 0 means not using the prosthesis at all during a week and 100 means it is used daily for >15 hours.^20^

### Objectives

The current study aimed to investigate the prevalence of events in the form of fractures and arthroplasties as well as the prosthetic use in patients treated with an OPRA implant.

### Statistical analysis

Data were analysed descriptively with frequency counts and percentages (n and %) for categorical variables. Software IBM SPSS V.29 was used for data analysis.

### Patient and public involvement

Patients and public were not involved in the study.

## Results

**Figure 1:**
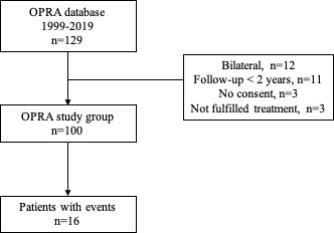
A flow diagram of study inclusion. OPRA, Osseointegrated Prosthesis for the Rehabilitation of Amputees.

A total of 100 patients met the inclusion criteria (figure 1). Patients were followed for a mean of 13.2 (±5.2) years after S2 and 56% had been followed for 10 years or more. The average age after completed surgical treatment (both S1+S2) was 44 years and the most common cause for TFA was trauma (n=70, 70%), followed by tumour (n=19, 19%) (table 1).

**Table 1:** Demographics in patients with, and without, complications, so-called events, in the lower limb after a BAP

	All patients n=100	No events in lower limb n=84	Events in lower limb n=16
Age, mean (SD)	59 (14)	58 (13)	64 (15)
Sex, n (%)			
Female	34 (34)	30 (36)	4 (25)
Male	66 (66)	54 (64)	12 (75)
Side, n (%)			
Left	49 (49)	46 (55)	3 (19)
Right	51 (51)	38 (45)	13 (81)
Residency, n (%)			
Europe, other than Sweden	39 (39)	33 (39)	6 (38)
Sweden, other region	52 (52)	45 (54)	7 (44)
Sweden, Western region	9 (9)	6 (7)	3 (19)
Cause for TFA, n (%)			
Trauma	70 (70)	61 (73)	9 (57)
Tumour	19 (19)	16 (19)	3 (19)
Embolism	3 (3)	3 (4)	0 (0)
Infection	8 (8)	4 (5)	4 (25)
ASA at surgery S1, n (%)			
1	42 (42)	37 (44)	5 (31)
2	26 (26)	22 (26)	4 (25)
3	2 (2)	2 (2)	0 (0)
Missing	30 (30)	23 (27)	7 (43)
BMI at BAP surgery, mean (SD)	26 (4)	26 (4)	25 (4)
Age completed BAP, mean (SD)	44 (13)	45 (13)	43 (13)
Years between TFA and S1, mean (SD)	11 (10)	10 (10)	13 (13)
Baseline prosthetic use score, mean (SD)	44 (36)	43 (35)	51 (41)
Latest prosthetic use score, mean (SD)	69 (35)	69 (35)	69 (37)
Cases of fixture reoperation, n (%)	13 (13)	10 (8)	3 (19)

ASA, American Society of Anesthesiologists ; BAP, bone-anchored prosthesis; BMI, body mass index; S1, stage 1; TFA, transfemoral amputation.

**Figure 2:**
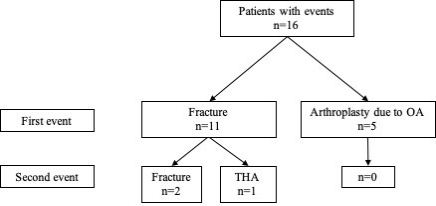
Distribution of postoperative events, grouped as either a fracture or arthroplasty due to OA, for patients with a transfemoral OPRA system. OA, osteoarthritis; OPRA, Osseointegrated Prosthesis for the Rehabilitation of Amputees; THA, total hip arthroplasty.

16 patients experienced a lower limb skeletal event, either arthroplasty surgery due to OA or a femur fracture (table 1, figure 2). There were more male patients in the group with lower limb skeletal events (n=12, 75%) compared with patients without lower limb events (n=54, 64%). Patients with these events had a higher baseline prosthetic usage, that is, increased use prior to BAP surgery, than those who later had no events (mean PUS of 51 vs 43. However, at the latest follow-up, patients reported on similar prosthetic usage regardless of lower limb events (mean PUS 69 in both groups). Revisions, that is, exchange, of the bone-anchored fixture was more common in the group with events (n=3, 19%) compared with those who did not experience any lower limb events (n=10, 8%).

### Patient with lower limb events

**Figure 3:**
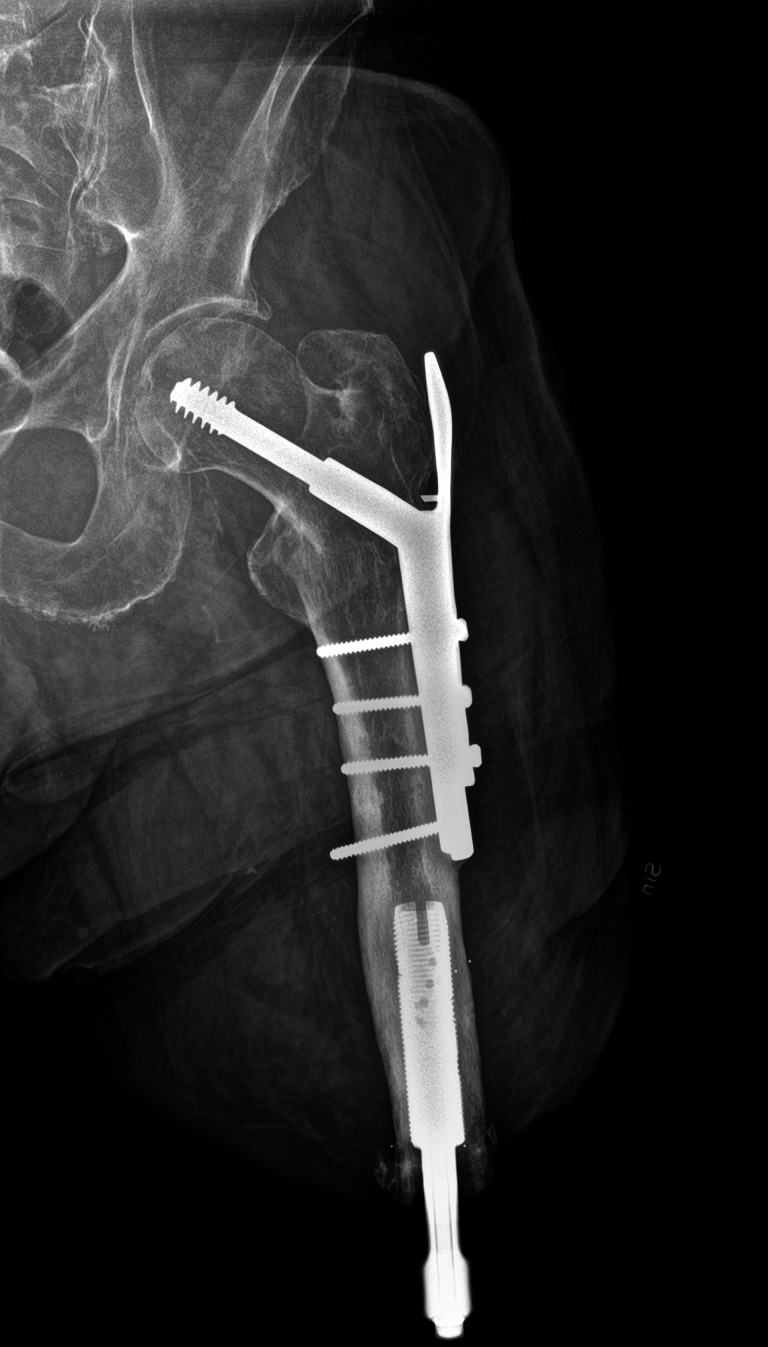
An example of a X-ray image of a pertrochanteric femur fracture fixated with osteosynthesis on the ipsilateral side as the BAP. BAP, bone-anchored prosthesis.

Patients with skeletal complications of the lower limb, referred to as ‘event’, had their TFA at a range of ages between 14 and 65 years and the time from amputation to event was from 1 to 43 years (table 2). A larger proportion of the patients (75%) were male. On average, patients experienced their event 6 (4.7 SD) years after their S2 and the most common first event was a fracture (n=11, 69%). All fractures were of the femur and on the ipsilateral side as the BAP fixture. Figure 3 illustrates an X-ray of a patient with BAP and ipsilateral osteosynthesis. The main reason for fracture was fall trauma (trauma mechanism was not described for all patients, however, at least 79% of fractures were due to falls). Patients with an arthroplasty due to OA as their first event (n=5) presented with contralateral OA most commonly of the knee (n=4, 80%). Three patients experienced yet another event after their first one in the form of a femur fracture (n=2) or total hip arthroplasty (THA) due to OA (n=1).

**Table 2:** Details on treatment in the 16 patients with lower limb complications, so-called ‘events’

Sex	Age amp (years)	Time to BAP (years)	Event	Time from BAP to event (years)	Side	Treatment	Event 2 and side	Treatment 2
M	46	13	fx	4	IL	ORIF	–	–
F	14	33	fx	13	IL	MIS	fx, IL	ORIF
M	21	18	fx	3	IL	ORIF	–	–
M	30	43	fx	13	IL	ORIF	–	–
F	46	12	fx	2	IL	ORIF	oa, CL	THA
M	63	2	oa	3	CL	TKA	–	–
M	32	1	fx	13	IL	MIS	fx, IL	ORIF
M	31	3	fx	1	IL	No surg	–	–
F	47	6	oa	13	CL	TKA	–	–
M	34	4	fx	10	CL	Extraction	–	–
M	25	31	oa	6	CL	TKA	–	–
F	17	5	fx	2	IL	No surg	–	–
M	37	23	oa	1	CL	THA	–	–
M	65	5	oa	3	CL	TKA	–	–
M	23	9	fx	8	IL	ORIF	–	–
M	60	1	fx	1	IL	HHA	–	–

Age amp, age when amputated; BAP, bone-anchored prosthesis; CL, contralateral; fx, fracture; HHA, hip hemiarthroplasty; IL, ipsilateral; MIS, minimally invasive surgery; oa, osteoarthritis; ORIF, open reduction, internal fixation; surg, surgery; THA, total hip arthroplasty; TKA, total knee arthroplasty.

One patient underwent a definitive extraction of the fixture. This was due to a fixture fracture and associated stress fracture of the femur. An additional two patients underwent a revision of their fixture, independent of their femur fracture. These two revisions occurred several years after the patients’ first event.

In regard to PUS, 50% of the patients (n=8) were not affected by their event and used their external prosthesis in the same manner as prior to their fracture/OA treatment at the latest recorded follow-up (table 3). Three patients (19%) reported an increased use of their prosthesis after their event and two patients (12.5%) reported a decrease.

**Table 3:** Patient-reported outcome, measured by prosthetic use score (PUS) in the group of patients with lower limb complications, so-called events

Patient	Baseline PUS	PUS before event*	PUS after event*	Difference PUS before and after event*	Latest recorded PUS
1	61	100	100	0	71
2	100	90	90	0	71
3	32	100	90	−10	71
4	90	90	90	0	90
5	0	52	90	+38	100
6	100	100	100	0	100
7	Missing	3	18	+15	18
8	90	Missing	Missing	Missing	100
9	71	90	Missing	Missing	90
10	4	100	Missing	Missing	100
11	90	100	100	0	90
12	0	23	0	−23	0
13	90	90	90	0	90
14	6	10	10	0	10
15	1	100	100	0	100
16	37	71	100	+29	10

Baseline PUS with a socket prosthesis before BAP treatment. PUS at follow-up closest possible before or after the event.

* Event is regarded as the first skeletal complication the patient experienced, if he or she experienced more than one postoperatively.

BAP, bone-anchored prostheses.

## Discussion

Little is known with regard to the long-term situation for patients with an OPRA system in terms of postoperative skeletal events of the lower limb. The current study aimed to evaluate postoperative lower limb events and found an incidence rate of 11% fractures and 6% arthroplasties due to OA in the years following the treatment. Two patients who had a fracture suffered a second fracture. However, the average prosthetic usage was the same in patients who had an event compared with those who did not.

Although the treatment team has had continuous contact with included patients and noted whether they have had complications at each follow-up, the fact that complete medical records were unavailable in the majority of patients is a limitation. There may be patients who have suffered lower limb complications unknown to the authors. Furthermore, there may be patients who have had issues with OA prior to their BAP treatment. Evaluating the presence of OA would have been more reliable if screening of symptoms of OA and radiographs had been a standard procedure before the OPRA treatment was initiated.

The number of patients in the current study is limited as BAP treatment is not vastly used. The number of events was too small to conduct any further rigorous analysis. Factors such as time from amputation to BAP treatment may affect the outcome and whether socket prostheses have been used in the meantime. Such factors would be interesting to analyse as well as rehabilitation details and motivation, as they most likely affect functional performance. Furthermore, it would have been of interest to have an age-matched control group to compare the prevalence of events in the BAP group to that of the general population.

The greatest strength of the present study lies in its volume of BAP patients and a follow-up time of at least 10 years among more than half of the study cohort and its long follow-up time. To the authors’ knowledge, it is the first study to describe the situation in regard to lower limb complications that patients with a TFA OPRA system face.

Örgel *et al* studied the rate of fractures after surgery with the endo-exo-prosthesis (EEP) system, a press-fit implant for amputees. Assuming Örgel *et al* found no bilateral fractures, comparing the rate of postoperative fractures, Örgel *et al* found a rate of 6% and Hoellwarth *et al* found a 5% rate, whereas the current study established a rate of 11%.^4 21^ Hoellwarth *et al* included BAPs in the femur, tibia and the upper extremity, making direct comparisons difficult. Additionally, the BAP systems of the three studies differ, and the OPRA system is constructed to spare the fixture in cases of excessive loading leading to fractures; hence, the abutment and abutment screw are more prone to fracturing. These can be exchanged without hampering the osseointegrated (fixture) component.^22^ However, the current study reports on a higher rate of femur fractures compared with the use of the EEP. The aim of the current study was not to compare the two systems, so the results should be interpreted with caution. Furthermore, Örgel *et al* employed a 12-month follow-up, whereas the current study had an average follow-up time of 13.2 years after S2. The average age of patients with EEP was 49 years compared with 59 years in the current study. The risk for femur fractures and OA increases with age, and the group of patients with events was older than those with no events (64 compared with 58 years of age). Higher age and especially longer follow-up time may explain the greater number of postoperative fractures in the current study.

Total knee arthroplasty (TKA) was most common in the group of patients who underwent arthroplasty surgery due to OA. Norvell *et al* reported on a higher rate of contralateral knee OA and knee pain in amputees compared with non-amputees.^23^ The increased prevalence of OA has previously been suggested to be due to the increased load amputees may put on the contralateral side after their amputation. Gait analyses have reported that amputees present with asymmetrical gait, and increased loads can be detected at the contralateral knee during weightbearing.^24^ In a recent systematic review, contralateral OA (66.7%) was found more common than ipsilateral.^25^ Surprisingly, few THAs due to OA were identified in the current study (n=1). In patients with TFA, OA of the hips is a known complication affecting both the amputated and contralateral side to a greater extent than non-amputees.^26^ The review by Walton *et al* suggests that the number of patients with THA should be higher, as THA (195/265) was more common than TKA (51/265).^25^ However, the current result should be interpreted with great caution as several patients may have been affected by hip OA and a full hip assessment with screening for OA symptoms and radiographs was not conducted.

An important finding in the present study was that prosthetic usage was similar regardless of whether an event had occurred. This is also supported by the findings of Örgel *et al* reporting that periprosthetic fractures in BAP treated amputees did not worsen functional outcome.^21^ In both studies, implant-preserving osteosyntheses of fractures were used, apart from one case in the current study which required implant extraction. Planning osteosynthesis of a hip fracture or periprosthetic fracture may be challenging as the implant may obstruct the preferred method of fracture fixation. Likewise, possibilities to treat OA with THA may be limited and pose a challenge for the surgical team, although there are successful reports of combinations of BAP and THA of the same side.^27 28^ Although BAP patients may require longer rehabilitation, physical function seems similar for patients with a BAP and THA compared with those undergoing THA.^25^

Nearly half of the fractures reported by Örgel *et al* were caused intraoperatively. No intraoperative fractures were identified in the current cohort, and all fractures of the current study were postoperative and mainly fall related (at least 79% were due to falls). Osteopenia and osteoporosis of the ipsilateral femur is a known complication after amputation,^18 26^ as is the increased risk for falls.^29^ Therefore, an elevated risk of postoperative fractures is not surprising. However, an increased risk for falls has been reported in association with the use of socket prostheses, where some falls have been perceived as a direct cause of the prosthesis as such.^30^ The current study does not include a proper fall evaluation. Future studies on BAPs are recommended to include information if a fall was while wearing or not wearing the prosthesis and details of prosthetic knee components. A BAP may offer better stability and thereby reduce the fall risk and perhaps increase prosthetic usage, in turn increasing bone mineral density due to increased weight-bearing.

The current study, including non-Scandinavian patients, reports on an increased incidence of hip fractures (11%) compared with the age-standardised hip fracture incidence in Scandinavia of 5%–6%, which is the highest in the world.^31^ When compared with the incidence of hip fractures in the general population, there are several factors to account for. Ethnicity, genetics, age and comorbidities greatly influence the risk and the overall risk for females is greater than for males.^32^ Interestingly, more males than females experienced lower leg events in the current study; this tendency was also present in the study by Örgel *et al.*^21^ The prevalence of THA or TKA in the general Swedish population is 3.2%, compared with 6% in the current study.^33^ The numbers are not directly comparable and are not age-matched; however, they suggest that there may be a higher prevalence of arthroplasties in BAP patients. This may be due to an increase in activity levels after BAP^34^ hypothetically also combined with a somewhat abnormal kinematic and kinetic gait.^35^

## Conclusions

Although larger comparative studies need to be conducted, patients with BAP may be at a higher risk for femur fractures and arthroplasty than the general population. This is important to consider when rehabilitating after BAP. Also, the presence of BAP may require special attention when planning ipsilateral osteosynthesis or arthroplasty. Prosthetic usage seems similar regardless of the patient has experienced a femur fracture or arthroplasty surgery of the lower limb. This is encouraging, however, further prospective studies with larger cohorts and control groups need to be conducted to compare functional outcomes and patient satisfaction in BAP patients with the general population.

## Data Availability

All data relevant to the study are included in the article or uploaded as supplementary information.
