# Supplementary material for: Non-infectious skeletal complications in the lower extremity after treatment with a transfemoral bone-anchored prosthesis: a retrospective observational study
Source: BMJ Open. 2026 Feb 24;16(2):e105415. doi: 10.1136/bmjopen-2025-105415 (PMC12933796; doi:10.1136/bmjopen-2025-105415)
Supplement: Supplementary file 1 [file bmjopen-2025-105415.reviewer_comments.pdf]

# PEER REVIEW HISTORY

BMJ Open publishes all reviews undertaken for accepted manuscripts. Reviewers are asked to complete a checklist review form and are provided with free text boxes to elaborate on their assessment. These free text comments are reproduced below.

## ARTICLE DETAILS

### Title (Provisional)

Noninfectious skeletal complications in the lower extremity after treatment with a transfemoral bone-anchored prosthesis - a retrospective observational study

### Authors

Svensson Malchau, Karin; Malchau, Henrik; Thomsen, Peter; Hagberg, Kerstin

---

## VERSION 1 - REVIEW

---

| | |
|---|---|
| **Reviewer** | **1** |
| **Name** | **Bafor, Anirejuoritse** |
| **Affiliation** | **Nationwide Children's Hospital** |
| **Date** | **04-Oct-2025** |
| **COI** | **None** |

---

Introduction:

The background and study objectives are presented clearly and are unambiguous.

Methods:

It would be helpful for the authors to clarify why patients with bilateral transfemoral amputations were excluded from the study. Additionally, there appears to be some inconsistency regarding the follow-up period: in the Results section, the mean follow-up is reported as 8.7 years postoperatively, while in the Methods section the average follow-up is stated as 13.2 years after the second staged procedure. Could the authors please reconcile this discrepancy?

Results:

The reported findings are valuable; however, further clarification regarding the follow-up time, as noted above, would improve transparency. Furthermore, were there any patients who required a secondary amputation during the follow-up period or were any of the surgical procedures included in this review secondary amputations?

Discussion:

The discussion would benefit from a clearer consideration of the implications of the findings, particularly in the absence of a control or comparison group.

Conclusion:

Without a comparative component, it is difficult to conclude that BAPs predispose patients to a higher risk of femoral fractures and arthroplasty. Given the descriptive nature of the study, the results should be interpreted with caution.

---

| | |
|---|---|
| **Reviewer** | **2** |
| **Name** | **Kuwahara, Yutaro** |
| **Affiliation** | **Toyohashi Municipal Hospital, orthopedic** |
| **Date** | **08-Dec-2025** |
| **COI** | **None** |

---

I am honored to have the opportunity to review this study.

General Comments:

The topic is certainly interesting, and complication rates at 5–10 years have already been reported in other studies. As the authors mention, investigating long-term outcomes is clinically meaningful. However, this study includes patients who were followed only for a few years after BAP implantation, and therefore it does not represent a fully long-term outcome assessment. Nevertheless, the manuscript seems to present long-term outcomes as a major strength of the study, which may lead to misunderstanding among readers. Please present this point clearly and avoid overstating the long-term nature of your results.

Abstract

Is the OPRA system an abbreviation for Osseointegrated Prostheses for the Rehabilitation of Amputees, or is it the name of a specific commercial product? Please clarify this point so that readers can better understand the terminology.

Although the abstract mentions long-term outcomes, the study includes patients with a minimum follow-up of only two years. Based on the information provided in the abstract alone, it is unclear whether true long-term outcomes were evaluated. Please either describe how long-term outcomes were assessed or remove the reference to long-term outcomes.

Methods

Please provide the full term when the abbreviation first appears in the manuscript.

Regarding the follow-up period, it would be more appropriate to report this information in the Results section. Since the aim of your study is to evaluate long-term outcomes, it might be helpful to include the range of follow-up durations and the proportion of patients who were followed for more than 10 years.

Results

The follow-up period is described in both the Methods and Results sections; however, the mean follow-up duration reported in each section differs. Could you clarify which value is correct?

Regarding Table 3, the PUS scores appear to show minimal change before and after the event in many cases. At what point after fracture treatment were the PUS scores after event obtained? For example, in a case such as the one shown in Figure 3, how did the PUS score change?

Discussion

In this study, for the patients who underwent arthroplasty due to osteoarthritis (OA), did the authors investigate whether OA was already present before BAP implantation or whether it developed after implantation? In addition, among the five cases that required arthroplasty, two patients had a considerable interval between amputation and BAP implantation. Does this suggest that the act of ambulating with a prosthesis after amputation, regardless of the type of prosthesis, might have been a risk factor? I would appreciate it if the authors could clarify their interpretation of these points.

In your manuscript, you state that surgical implantation of a prosthesis may increase the likelihood of decreased bone mineral density (BMD) in the contralateral limb. Did you measure BMD or any related parameters in this study?

According to Table 2, half of the patients who experienced fractures or required arthroplasty after BAP insertion experienced these events within 3 years postoperatively. In contrast, only 2% underwent joint replacement and 5% sustained fractures beyond 5 years, which is nearly equivalent to the rates reported for the general population. This pattern may indicate a higher incidence of mechanical complications within the first 3 years after surgery. If possible, please include a discussion of these findings.

Figure legends

Please revise the figure legends so that the content is clearly understandable to readers based solely on the figures and their descriptions. Additionally, for Figure 3, it would be more informative to include radiographic images from the pre-fracture, post-fracture, and postoperative stages to improve clarity for the readers.

I would like to thank you again for giving me this valuable chance.

---

## VERSION 1 - AUTHOR RESPONSE

Reviewer: 1
Dr. Anirejuoritse Bafor, Nationwide Children's Hospital
Comments to the Author:
Introduction:
The background and study objectives are presented clearly and are unambiguous.

Methods:
It would be helpful for the authors to clarify why patients with bilateral transfemoral amputations were excluded from the study. Additionally, there appears to be some inconsistency regarding the follow-up period: in the Results section, the mean follow-up is reported as 8.7 years postoperatively, while in the Methods section the average follow-up is stated as 13.2 years after the second staged procedure. Could the authors please reconcile this discrepancy?

Thank you for your comment. The reason for exclusion is now added in the methods section. Regarding follow-up time, patients were followed for a mean of 13.2 +/- 5.2 years after S2, thank you for helping us clarify this.

Results:
The reported findings are valuable; however, further clarification regarding the follow-up time, as noted above, would improve transparency. Furthermore, were there any patients who required a secondary amputation during the follow-up period or were any of the surgical procedures included in this review secondary amputations?

Thank you for your question. No patients required a secondary amputation during the follow-up. Patients may have undergone secondary amputations prior to their BAP. However, this is unknown to us as the OPRA database does not contain this information and medical records on amputation surgery were not obtained. Only surgical procedures after BAP surgery were reviewed.

Discussion:
The discussion would benefit from a clearer consideration of the implications of the findings, particularly in the absence of a control or comparison group.

We agree that the study would have benefitted from a matched control group. In the absence of this, we tried to find the incidence of hip fractures and arthroplasty due to OA in the general population as described in the discussion. Based on these numbers, the risk for a hip fracture or arthroplasty due to OA was higher in patients with BAPs. However, as you point out, concluding that BAPs may increase the risk of a femoral fracture or arthroplasty might be a bit too bold without having performed a comparative study.

Conclusion:
Without a comparative component, it is difficult to conclude that BAPs predispose patients to a higher risk of femoral fractures and arthroplasty. Given the descriptive nature of the study, the results should be interpreted with caution.

The conclusion has been revised to highlight the need for comparative studies in order to fully establish risk of femoral fractures and arthroplasty after BAP surgery.

Reviewer: 2
Dr. Yutaro Kuwahara, Toyohashi Municipal Hospital
Comments to the Author:

I am honored to have the opportunity to review this study.

General Comments:
The topic is certainly interesting, and complication rates at 5–10 years have already been reported in other studies. As the authors mention, investigating long-term outcomes is clinically meaningful. However, this study includes patients who were followed only for a few years after BAP implantation, and therefore it does not represent a fully long-term outcome assessment. Nevertheless, the manuscript seems to present long-term outcomes as a major strength of the study, which may lead to misunderstanding among readers. Please present this point clearly and avoid overstating the long-term nature of your results.

Thank you for your comment. As pointed out, the study does include patients who were followed for a shorter period of time as well as patients who were followed for 20+ years. To avoid misleading the reader we have revised our manuscript accordingly.

Abstract
Is the OPRA system an abbreviation for Osseointegrated Prostheses for the Rehabilitation of Amputees, or is it the name of a specific commercial product? Please clarify this point so that readers can better understand the terminology.

This is the name of a specific commercial system. This has now been clarified in the manuscript.

Although the abstract mentions long-term outcomes, the study includes patients with a minimum follow-up of only two years. Based on the information provided in the abstract alone, it is unclear whether true long-term outcomes were evaluated. Please either describe how long-term outcomes were assessed or remove the reference to long-term outcomes.

Thank you for the comment. This has now been removed from the abstract in order not to mislead the reader.

Methods
Please provide the full term when the abbreviation first appears in the manuscript.

Thank you for this observation. We have now revised the manuscript and hope that all abbreviations are explained.

Regarding the follow-up period, it would be more appropriate to report this information in the Results section. Since the aim of your study is to evaluate long-term outcomes, it might be helpful to include the range of follow-up durations and the proportion of patients who were followed for more than 10 years.

This has now been revised and is removed from the methods section and is in the results section. The proportion of patients followed for more than 10 years was 56%.

Results
The follow-up period is described in both the Methods and Results sections; however, the mean follow-up duration reported in each section differs. Could you clarify which value is correct?

Regarding follow-up time, patients were followed for a mean of 13.2 +/- 5.2 years after S2, thank you for helping us clarify this.

Regarding Table 3, the PUS scores appear to show minimal change before and after the event in many cases. At what point after fracture treatment were the PUS scores after event obtained? For example, in a case such as the one shown in Figure 3, how did the PUS score change?

PUS scores were evaluated at every standardised follow-up. Therefore, we chose to analyse the PUS score at the following standardised follow-up after an event. Knowing that the time between an event and following follow-up could differ between patients we also analysed the latest recorded PUS to see if an event contributed to lower PUS in a longer term perspective.
For the patient in Figure 3, PUS was 100 before fracture (at 3 year follow-up) and 100 after fracture (5 year follow-up).

Discussion
In this study, for the patients who underwent arthroplasty due to osteoarthritis (OA), did the authors investigate whether OA was already present before BAP implantation or whether it developed after implantation? In addition, among the five cases that required arthroplasty, two patients had a considerable interval between amputation and BAP implantation. Does this suggest that the act of ambulating with a prosthesis after amputation, regardless of the type of prosthesis, might have been a risk factor? I would appreciate it if the authors could clarify their interpretation of these points.

Thank you for this comment. Since the study cohort consists of patients who are treated at other hospitals, in Sweden, and internationally, their medical journals were not available to us. Hence, there may be patients who have radiographic OA unknown to us, and, as pointed out, OA may have been present before BAP implantation in some patients. We believe that ambulating as an amputee as such is a risk factor for OA as all weight is put on the contralateral side. Wearing a prosthesis may lessen the burden as weight is distributed somewhat more evenly.

In your manuscript, you state that surgical implantation of a prosthesis may increase the likelihood of decreased bone mineral density (BMD) in the contralateral limb. Did you measure BMD or any related parameters in this study?

Theoretically, increased weight-bearing, which using a prosthesis implies, should contribute to increased bone mineral density, however, no measurement of BMD was conducted. However, the comment is of importance and this measure will be incorporated in our future studies.

According to Table 2, half of the patients who experienced fractures or required arthroplasty after BAP insertion experienced these events within 3 years postoperatively. In contrast, only 2% underwent joint replacement and 5% sustained fractures beyond 5 years, which is nearly equivalent to the rates reported for the general population. This pattern may indicate a higher incidence of mechanical complications within the first 3 years after surgery. If possible, please include a discussion of these findings.

Thank you for this reflection. This could be a possibility, however, mechanical complications have previously been reported more frequent after 5 years from surgery with an OPRA implant (Hagberg  K, Ghasemi Jahani SA, Omar O, Thomsen P. Osseointegrated prostheses for the

rehabilitation of patients with transfemoral amputations: A prospective ten-year cohort study of patient-reported outcomes and complications. J Orthop Tranlat. 2022 Oct 20;38:56-64)

Figure legends
Please revise the figure legends so that the content is clearly understandable to readers based solely on the figures and their descriptions. Additionally, for Figure 3, it would be more informative to include radiographic images from the pre-fracture, post-fracture, and postoperative stages to improve clarity for the readers.

The figure legends have now been revised. The medical records for the patient in Figure 3 have been reviewed once more. However, the X-ray images from pre-fracture and fracture period are of such old date that they are no longer saved in our medical record system. Otherwise we agree that this would have been illustrative.

I would like to thank you again for giving me this valuable chance.

Reviewer: 1
If you have selected 'Yes' above, please provide details of any competing interests.: Not applicable

Reviewer: 2
If you have selected 'Yes' above, please provide details of any competing interests.: Not applicable.

## VERSION 2 - REVIEW

| | |
|---|---|
| **Reviewer** | **1** |
| **Name** | **Bafor, Anirejuoritse** |
| **Affiliation** | **Nationwide Children's Hospital** |
| **Date** | **29-Jan-2026** |
| **COI** | |

No further comments at this time

| | |
|---|---|
| **Reviewer** | **2** |
| **Name** | **Kuwahara, Yutaro** |
| **Affiliation** | **Toyohashi Municipal Hospital, orthopedic** |
| **Date** | **29-Jan-2026** |
| **COI** | |

I am honored to have the opportunity to review this study.

Thank you for your accurate replies to each of our comments.   Your research is now more attractive and understandable to readers. Your revised manuscript will be evaluated as worthy of publication.

I would like to thank you again for giving me this valuable chance.
